# Feasibility of Implementing the Advanced Dementia Prognostic Tool in Nursing Home Assessments

**DOI:** 10.1093/geroni/igaf122.807

**Published:** 2025-12-31

**Authors:** Susanny Beltran, Latarsha Chisholm, Vittoria Figueiro

**Affiliations:** University of Central Florida, Orlando, Florida, United States; Univeresity of Central Florida, University of Central Florida, Florida, United States; University of Central Florida, Orlando, Florida, United States

## Abstract

Identifying hospice eligibility for nursing home residents with advanced dementia remains inconsistent, leading to delays in appropriate end-of-life care. The Advanced Dementia Prognostic Tool (ADEPT) has the potential to standardize prognostic assessments, but its feasibility for routine nursing home integration is unclear. This study evaluated ADEPT’s feasibility across four monthly assessment cycles in a skilled nursing facility. Data were collected on completion rates, documentation consistency, staff adherence to protocol, and perceived barriers and facilitators. Compliance with recommended actions based on ADEPT scores was assessed, and deviations were analyzed for justification and impact on care planning. ADEPT was successfully integrated into Minimum Data Set (MDS) evaluations, with compliance improving over time. Of the 48 assessments due, 34 were submitted in compliance. However, staff frequently omitted score documentation, citing concerns over regulatory scrutiny and potential repercussions. Uncertainty regarding score interpretation underscored the need for a clear threshold to guide decision-making. Barriers included staff training needs and regulatory apprehension, while facilitators included workflow alignment and structured interdisciplinary communication. Staff preferred using ADEPT to support goals-of-care discussions at family conferences rather than as an automatic hospice referral trigger. Findings demonstrate that ADEPT is a feasible addition to routine nursing home assessments, improving consistency in hospice eligibility identification. However, additional strategies are needed to support consistent score documentation, ensure regulatory confidence, and integrate ADEPT into structured decision-making processes. Future research should focus on best practices for incorporating ADEPT scores into clinical workflows to enhance goals-of-care discussions and promote care concordance with residents’ needs and preferences.

